# The Management of Some Obstetric Emergencies
*A paper read before the Bristol Medico-Chirurgical Society, 11th January, 1939.


**Published:** 1939

**Authors:** Gilbert I. Strachan

**Affiliations:** Professor of Obstetrics and Gynæcology in the University of Wales; Obstetrician and Gynæcologist to Cardiff Royal Infirmary


### THE MANAGEMENT OF SOME OBSTETRIC EMERGENCIES.*

BY

GILBERT I. STRACHAN, M.D., F.R.C.P., F.R.C.S., F.R.C.O.G.,

*Professor of Obstetrics and Gynæcology in the University of Wales; Obstetrician and Gynæcologist to Cardiff Royal Infirmary.*

---

IN recent years increasing stress has been laid by speakers and writers on the importance of ante-natal care in the preventive aspect of obstetrics, and it has come to be believed that by such means the great majority of obstetric emergencies can be avoided. That this is not fact has been the cause of many discussions on such subjects as "Why are we dissatisfied with the results of ante-natal observation," when the fault is not with the results of ante-natal observation but with those who built extravagant hopes and anticipations on such care. In the first flush of the recent outcry about maternal mortality the custom was to blame the general practitioner for every fault in our obstetric services: but later, when the nation-wide institution of ante-natal clinics had failed materially to reduce that mortality, it became the fashion to blame the amount or the kind of supervision received. It is not my intention to discuss the scope

* A paper read before the Bristol Medico-Chirurgical Society, 11th January, 1939.

of ante-natal supervision with its manifest advantages and definite limitations, except to emphasize this point; that no matter how complete such ante-natal supervision may be, emergencies, unexpected and often of a grave nature, are bound to occur from time to time in obstetric practice, and that the obstetrician must be properly armed with knowledge and with equipment to deal with such emergencies. It is this emergency aspect of obstetrics that I wish to discuss with you this evening. A preliminary point, not usually emphasized, is that the degree of emergency which a particular case presents depends not only on the clinical condition of the case, but almost equally on the general conditions and surroundings of the patient.

If we take three kinds of such conditions it will illustrate what I mean. Thus a case of ante-partum hæmorrhage occurring in a well-equipped hospital may cause little upset: it can be kept under observation and any necessary intervention can be carried out at the appropriate time and under proper conditions. But a similar complication occurring in a house in a town is much more upsetting. Removal to hospital or nursing home is indicated, and this, together with the family upset, represents a formidable effort and consumption of time. Again, in a cottage isolated in the country, the matter may be very grave. There is the distance, often long, to be traversed, the unsuitable domestic conditions for doing anything, and the difficulty and danger of transport, so that it may be for the best not to remove the patient, and the practitioner is faced with the full responsibility of a major obstetrical crisis. The importance, then, of the attendant conditions is great and cannot be over-emphasized, and it may be necessary again to refer to this aspect of the matter.

The possible obstetrical emergencies are very numerous, and I shall confine myself to those most commonly encountered and shall endeavour to discuss the means we find most useful in dealing with them according to the surrounding conditions. The hæmorrhages represent some of the most important and urgent of these emergencies and call for special discussion.

### Hæmorrhage.

*Ante-partum hœmorrhage* is mostly due to placenta prævia, and no condition may be more dramatic or call for more calm judgement. The first thing to be remembered is that shock is as great a danger in this condition as is the actual loss of blood, and in many cases the degree of shock present may be profound and bear no ratio to the amount of blood lost. The indication, therefore, is to manipulate the patient as little as possible, and with the greatest gentleness: and it is found in practice that in the vast majority of cases minor measures suffice to control the condition. In the first place, is it necessary to do anything active at all? If the bleeding is not severe, and if the contractions are good, pressure on the separated placenta by the presenting part is likely to solve the difficulty, and merely with observation the labour may be completed, possibly with the aid of low forceps. If this is not effective, it is not sufficiently recognized how great benefit may be obtained merely by rupturing the membranes: this is a simple manipulation which allows the head to be directly pressed on the bleeding placental site, so that, in the great majority of cases, the hæmorrhage is controlled. If this is still unsatisfactory, Willett's forceps can be applied to the fœtal scalp if the cervix admits two fingers. If the head is steadied this can usually be done without anæsthesia, and if a good grip of the scalp be obtained, traction by a one-pound weight is just sufficient to keep up pressure and to control the bleeding.

Only if these measures fail need version be performed, and this requires the one and only anæsthetic to be employed. With a leg brought down in this way, in a marginal case the bleeding will be absolutely controlled and delivery should soon occur. Vaginal packing may be required in some cases, but in general practice should always be avoided. It must be remembered that vaginal packing, to be effective, is a major obstetrical operation : anæsthesia and antisepsis are necessary, also very many yards of sterile gauze if the fornices are to be properly packed, and such conditions are only to be obtained properly in hospital. Usually an insufficient amount of material is packed into the vagina, the main result of which is to predispose to sepsis. If the patient has to be sent to hospital the best thing is to give morphia gr. $\frac{1}{4}$ and to do nothing else, not even to examine.

*Post-partum Hæmorrhage* is another ever-present danger, and may be most grave. It is usually supposed to follow prolonged and exhausting labour, inertia, or twin pregnancy : but in many cases it follows a perfectly normal labour, and in other cases, when it might be expected, it fails to appear. As the condition is mostly atonic the first and main thing is to control the fundus, and this may sometimes be difficult.

Thus if the uterus has rapidly distended, its limits may be very vague, and a grab at the abdomen say in the region of the umbilicus may result in grasping the body of the uterus below the fundus, and so is useless. To make sure of grasping the fundus the hand should be sunk deeply into the slack abdomen just below the ensiform and carried down towards the umbilicus: only in this way can the fundus be with certainty identified and controlled, and often a gush of retained blood will indicate that this has been accomplished. Once this has been done, and an injection of ergometrine or pituitary extract given, the next indication is to keep the patient absolutely quiet and at rest. It will follow, therefore, that such cases should never be transferred to hospital. The responsibility has to be faced on the spot and with such simple measures the vast majority of cases will be successfully controlled: in only a very few will such measures as bimanual compression be necessary.

### OCCIPITO-POSTERIOR PRESENTATION.

The occipito-posterior position of the vertex represents a complication and an emergency that is painfully familiar to many of us, and it has well earned the name of the bugbear of obstetrics. Much has been written regarding the causation of this condition and much conflicting advice has been given regarding its treatment. It is of the latter that I wish to speak, and I would like to emphasize the following points:—

(1) So long as progress in labour is being made interference is contra-indicated. With a not too large head spontaneous birth may occur with the occiput posterior, and this does not always cause undue damage to the soft parts, and is preferable to the most expert interference.

(2) When progress has ceased the head has to be turned to make the occiput anterior, and this can always be done manually. Rotation by forceps is quite unnecessary and may cause much laceration of the vagina and the fœtal face.

In rotating a posterior occiput one of the most important factors is not only anæsthesia, but deep surgical anæsthesia: with this the parts are relaxed and rotation is easy, but if the patient merely has a "whiff" muscular resistance may make rotation impossible. Next, the whole hand should be introduced up the vagina and the position confirmed by feeling the ear: the head should then be disregarded and the hand carried upwards until the index finger is behind the anterior shoulder. If necessary the head should be lifted up out of the pelvis for this to be accomplished. Then with a rotating movement the shoulder is carried a full half circle across the anterior segment of the pelvis, and the head, which automatically follows, then becomes occipito-anterior and will remain so.

A trial of this manœuvre, which was described by Lamond Lackie several years ago, will demonstrate its great ease *provided that anæsthesia be sufficiently deep.* It is surprising that it has not been universally adopted to the exclusion of the difficult and uncertain bimanual method, in which, so often, only the head is rotated and slips back whenever the hand is removed.

(3) When the head has been successfully rotated, forceps extraction is usually indicated, as the patient has mostly had a long labour. But in some cases fresh moulding in the anterior position may be necessary before this can be safely accomplished. Therefore, if extraction is not easy, if there is that sense of stuck resistance, the forceps should be removed and the head allowed more time for moulding. It has been my personal experience that the observance of these points has robbed this complication of most of its terrors.

### Laceration.

It may seem strange that I discuss so common an event as a *ruptured perineum* with you, but there are several points in the treatment of this common emergency that are of importance. The more thoroughly such a laceration is repaired at the time of occurrence the greater comfort will the patient have, and the less will be the probability of future prolapse. The first thing is clearly to define the limits and the depth of the laceration, and this is difficult when the parts are obscured by blood descending from the uterus. If a handful of swabs squeezed out of lotion be pressed up to the top of the vagina this will form a pad and so the field will be left clear and the limits of the laceration may then be seen. The operation of repair can then only be properly performed if there is a good strong direct light on the part, and this is easily secured by a large electric torch which should form an essential part of every practitioner's outfit. With such a light the deep and superficial suturing can be carried out with exactness and to whatever depth or extent necessary. These small points make all the difference between a perineal body properly repaired and the blind insertion of sutures here and there in a blood-soaked field in the dark.

### SHOCK.

Obstetric shock is an alarming condition when it occurs, and it usually does so unexpectedly. In antepartum hæmorrhage, as mentioned, and after prolonged labour, especially with much manipulation, a varying amount of shock may be present. But the condition I have in mind is one in which the labour has been normal in course and duration, but after which, for no obvious clinical reason, the patient collapses with profound pallor and faint, rapid pulse. Thus in one case in my experience the patient collapsed immediately after the birth of the child, and was so ill that it was only two days later that the placenta could be removed. The cause of this condition is not at all clear, but in some cases it is supposed to be due to partial rupture of the uterus, while in other it is held to be an anaphylactic phenomenon due to the administration of pituitary. The best line of treatment in such cases is certainly sedative. Everyone except the attendants should be excluded, the room should be darkened, and morphia gr. $\frac{1}{4}$ should be given. With such treatment, aided if possible by a rectal saline, recovery is usually complete and in some cases surprisingly rapid. Cardiac stimulants, pituitary or adrenaline are best avoided as they may overstimulate an exhausted sympathetic nervous system.

### ILEUS.

*Paralytic ileus* is a rare complication after labour, and usually follows a difficult instrumental confinement. The condition is alarming. Within an hour the abdomen may be greatly distended and tympanitic and the patient so gravely ill that urgent attention is necessary. The best thing here is to give repeated molasses enemata with pituitary, and it will be found that in most cases flatus and later fæcal matter will be passed and the abdominal distension will subside.

### Sepsis.

Puerperal sepsis is not usually of rapid onset, and its treatment is usually provided for by local health authorities: but in some cases the onset and course are very rapid and the case has to be treated at home under emergency conditions. These cases fall into two main groups: in the majority of cases there has been previous manipulation during labour, and in such cases the infecting organism may be the hæmolytic streptococcus, the *bacillus coli*, or the staphylococcus: in the minority of cases sepsis develops after a perfectly normal untouched delivery or even when the baby has been born before the arrival of the attendant. The infecting organism is usually the hæmolytic streptococcus carried from the throat of an attendant, and the condition is usually very serious, indicating an inherent lack of resistance in the patient. The prognostic points, such as time of onset, degree of anæmia, amount of pyrexia, rapidity of pulse, and so on, are well known: but in many cases these points are indeterminate and in no class of case is it more difficult to give a prognosis for the individual patient. In my own experience I find the following of most prognostic significance:—

(1) The time of onset: in general the earlier the onset the worse is the outlook.

(2) The degree of anæmia: the more marked is this the worse the outlook.

(3) The condition of the tongue: a dry, foul tongue usually indicates a fatal conclusion, and a moist red one a good outlook.

(4) The condition of the epigastrium: if there is tender rigidity here, generalized peritonitis is present and the outlook all but hopeless.

As regards treatment certain general points have to be remembered. Thus the shoulders should be elevated to allow of free pelvic drainage. If the patient lies flat, foul lochia may accumulate in the posterior fornix and the condition be aggravated. Again, the uterus should be left alone as far as possible: any unnecessary manipulation, such as forceful bimanual examination or curettage, is likely still further to disseminate septic material into the circulation. If it is suspected that a piece of placenta has been retained *gentle* exploration with the gloved finger may be carried out, but nothing more than this should be done. For the same reason intra-uterine douching is contra-indicated, as it tends to make the septic process extend upwards. An occasional gentle vaginal lavage may be given merely to wash away discharge and so to improve the patient's general comfort. The general strength should be conserved by appropriate easily-digested food, and by stimulants, and everything possible should be done to increase the general comfort.

Many drugs have been advocated and used in the treatment of puerperal sepsis, but they have been largely superseded in recent years by Prontosil and its various sulphonamide derivatives. The effectiveness of this drug is now hardly in doubt, but the advantage of such a general background of treatment as we have sketched cannot be over-emphasized. Prontosil cannot always work miracles, and it will not cure every case. Some cases of puerperal sepsis, especially those with general peritonitis, are fatal from the onset, and any form of treatment is useless. The dosage of Prontosil must be accommodated to the clinical condition, but our object should be to push the drug until the system is saturated, after which it can be reduced. Thus in a severe case 15 grains of Prontosil album should be given by mouth three times daily, and this should be reduced only as the pyrexia subsides, while for five days after the temperature is normal $7\frac{1}{2}$ grains should be given once daily. In any event a certain degree of cyanosis is apt to develop during the treatment: but if sulphur compounds, such as magnesium sulphate, are given at the same time, the condition of sulphæmo-globinæmia may develop, and an extreme degree of cyanosis occur, which may be terrifying in its intensity. But with a temporary reduction or withdrawal of the drug this condition soon disappears. This accidentally discovered drug, whose exact method of action is not yet clearly understood, represents a most important addition to our armament in the fight against puerperal sepsis, and it is mainly as the result of this that our maternal mortality has now fallen to the low level of 3.13 per 1,000 births.

### Retroversion.

The *incarcerated retroverted gravid uterus* which may be encountered about the third month may form an urgent emergency most distressful to the patient. Many methods of treatment, such as putting the patient in the knee-chest position, traction on the cervix with volsella, and upward pressure in the posterior fornix, have been advocated, but they are uncertain and can mostly be disregarded; while rough manipulation may produce abortion. Again the importance of deep anæsthesia is found, and if the bladder be first completely emptied by catheter the uterus can usually be easily mobilized upwards by simple pressure and retained in position by a pessary for three weeks. But the best method of all, which requires no anæsthesia, is simply to empty the bladder completely and *slowly* by a narrow rubber catheter. In this way the bladder may be half emptied at first, and it may take several further insertions before it is completely emptied. By these means alone the uterus will be found in practically every case to have spontaneously regained its normal position, and if the bladder is not allowed to get distended again the probability of recurrence is very slight.

I have purposely confined my remarks to some of the more common obstetrical emergencies, and I have discussed these largely as I have found them, and from my personal clinical experience, so that I have omitted all reference to figures and percentages. Again, I have looked at the conditions from the point of view of domestic practice with all its limitations, so that such invaluable adjuncts to treatment as blood transfusion or Cæsarean section in appropriate cases of ante-partum hæmorrhage have not been discussed; and it has been supposed that a well-equipped hospital is not at hand, and that the patient has to be treated at home. Finally, from what has been said it will be clear that he who knows his case best before confinement and who interferes the least during confinement will experience the minimum of obstetrical emergencies, and will be able to meet them to the best advantage.

